# Supplementary material for: YAP Promotes Cell Proliferation and Stemness Maintenance of Porcine Muscle Stem Cells under High-Density Condition
Source: Cells. 2021 Nov 8;10(11):3069. doi: 10.3390/cells10113069 (PMC8621012; doi:10.3390/cells10113069)
Supplement: Supplementary file 1 [file cells-10-03069-s001.zip › cells-1420680-supplementary.pdf]

Supplementary Figure S1

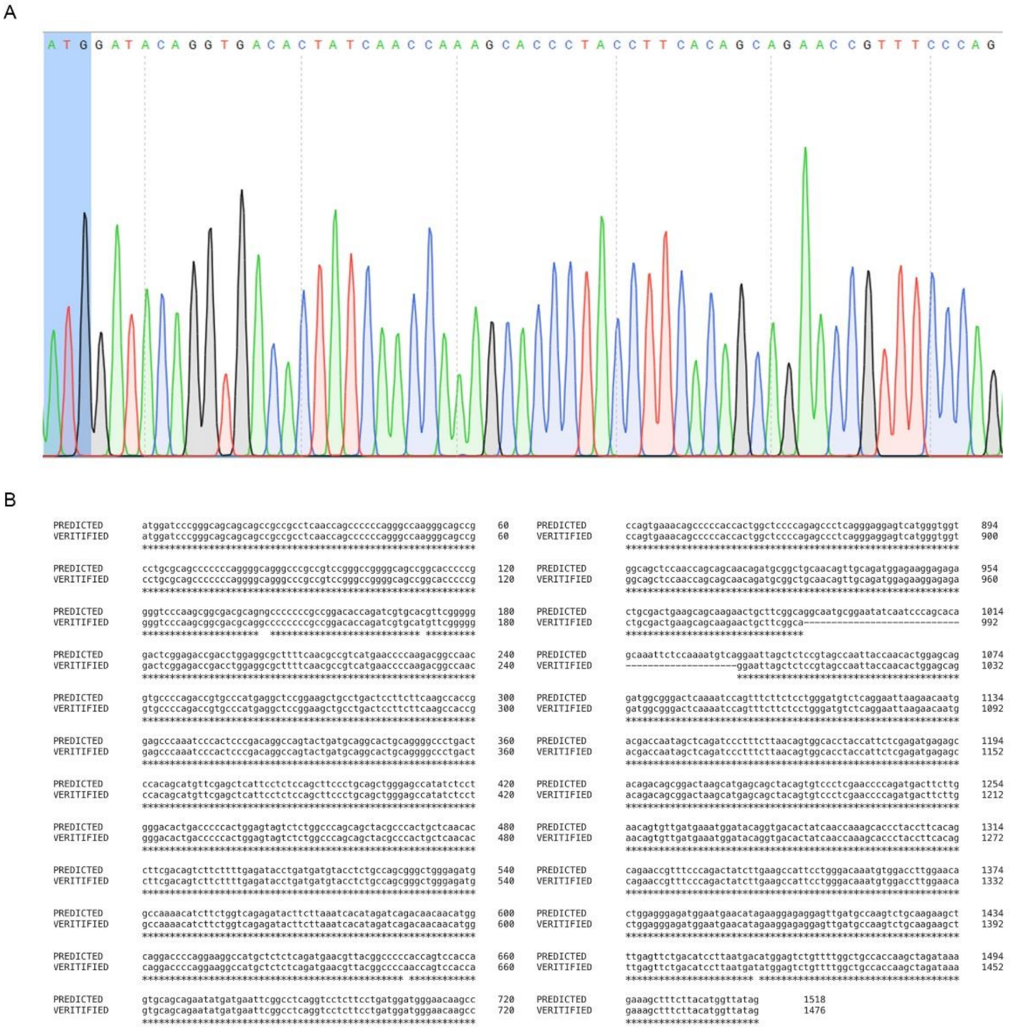

Figure S1. Cloning of the full-length of porcine *YAP1* CDS. (A) The porcine *YAP1* CDS was obtained by PCR amplification and verified by Sanger sequencing. (B) Confirmed porcine *YAP* CDS sequences exhibit 96% similarity to predicted sequenced in NCBI database (XM_021062706).
